# Efficacy of Office-Based Salivary Ductal Steroid Irrigation for Managing Post-Irradiation Xerostomia in Head and Neck Cancer Patients: A Retrospective Study

**DOI:** 10.3390/biomedicines12051033

**Published:** 2024-05-08

**Authors:** Yen-Chun Chen, Nguyen-Kieu Viet-Nhi, Luong Huu Dang, Chin-Hui Su, Shih-Han Hung

**Affiliations:** 1 Graduate Institute of Medical Sciences, College of Medicine, Taipei Medical University, Taipei 110301, Taiwan; cd30617@gmail.com; 2 Department of Otolaryngology, Taipei Medical University Hospital, Taipei 110301, Taiwan; 3 Department of Otolaryngology, School of Medicine, College of Medicine, Taipei Medical University, Taipei 110301, Taiwan; su.chin@msa.hinet.net; 4 International Master/Ph.D. Program in Medicine, College of Medicine, Taipei Medical University, Taipei 110301, Taiwan; drvietnhi@gmail.com; 5 Department of Otolaryngology, Faculty of Medicine, University of Medicine and Pharmacy at Ho Chi Minh City, Ho Chi Minh City 700000, Vietnam; luonghuudang167@gmail.com; 6 Department of Otolaryngology, Mackay Memorial Hospital, Taipei 104217, Taiwan; 7 Department of Otolaryngology, Wan Fang Hospital, Taipei Medical University, Taipei 116079, Taiwan

**Keywords:** xerostomia, head and neck cancer, salivary ductal irrigation, salivary amylase, chronic sialadenitis

## Abstract

Post-irradiation xerostomia remains a significant quality of life concern for patients with head and neck cancers. Conventional therapies offer limited effectiveness. This study aims to investigate the therapeutic potential of office-based salivary ductal steroid irrigation in patients with post-irradiation xerostomia. This single-center observational study recruited 147 head and neck cancer patients suffering from post-irradiation xerostomia between November 2020 and October 2022. All included subjects received at least one round of successful salivary ductal cannulation and irrigation. The primary measure of efficacy was improvement in subjective xerostomia and objective salivary amylase levels. A logistic regression was employed to evaluate factors affecting treatment responsiveness. The response rate among nasopharyngeal cancer (NPC) patients was 74.8%, and that among non-NPC cancer was 65.6%, without significant intergroup differences. The statistical analysis revealed no significant influence of age, gender, or disease stage on treatment responsiveness. Post-treatment salivary amylase levels were significantly higher in responsive non-NPC patients. In conclusion, salivary ductal steroid irrigation emerged as a promising therapeutic modality for the management of post-irradiation xerostomia in head and neck cancer patients. While no explicit factors were predictive of responsiveness, the high rate of symptom improvement suggests that this therapy may be a viable alternative for patients that are refractory to standard treatments.

## 1. Introduction

Post-irradiation xerostomia is one of the major complications in managing head and neck cancer patients. This dry mouth disorder strongly increases the risk of developing dental caries, and leads to difficulties with chewing, swallowing, and sleep disorders, significantly impacting patients’ quality of life [1,2]. Traditionally, post-irradiation xerostomia is managed by sialagogues stimulations, the use of artificial saliva, or by the administration of pilocarpine [3,4,5,6]. However, frequent cholinergic side effects such as sweating, nausea, emesis, diarrhea, and increased urinary frequency after taking antimuscarinic agents have limited their long-term usage [7,8]. As blockage of salivary flow, ductal strictures, or fibromucinous debris might develop after the irradiation, the salivary gland is in a status similar to chronic sialadenitis, and therapeutic maneuvers, including intraductal irrigation, might play an important role in the management of these patients [9,10].

The technique of salivary ductal irrigation has been established and utilized for the treatment of numerous salivary diseases, including chronic obstructive sialadenitis [11], chronic recurrent parotitis [12], and xerostomia in Sjogren’s syndrome [13,14,15]. It has been reported that sialendoscopy increased saliva secretion and reduced xerostomia for up to 60 weeks in Sjögren’s syndrome patients [16]. Up to 84% of subjective improvement in dry mouth following intraductal irrigation of salivary glands was reported by using only saline [17]. The effect of salivary ductal steroid irrigation on head and neck cancer patients with post-irradiation xerostomia remains largely unknown.

Considering the fact that there are no gold-standard techniques or protocols for salivary ductal irrigation currently, in our previous preliminary study, we demonstrated that simple office-based salivary ductal irrigation could be performed as an outpatient procedure to alleviate glandular swelling or xerostomia in patients with Sjogren’s syndrome, postradiotherapy sialadenitis, or post-RAI sialadenitis, and the procedure can be considered to be an alternative management approach for patients that are refractory to conventional strategies [18]. Specifically, approximately 60% (6/10) of post-radiotherapy xerostomia patients who underwent an average of 4.0 ± 1.1 monthly sessions of salivary ductal irrigation therapy showed responsiveness.

In this investigation, our primary objective is to assess the therapeutic efficacy of uncomplicated office-based salivary ductal steroid irrigation coupled with ductal dilatation in individuals with head and neck cancer experiencing post-irradiation xerostomia. Our hypothesis posits that this form of irrigation therapy will lead to both subjective clinical enhancements and objective improvements in salivary amylase levels.

## 2. Materials and Methods

### 2.1. Study Population and Design

This retrospective study was conducted at the Department of Otolaryngology, Taipei Medical University Wan Fang Hospital in Taipei, Taiwan. The Institutional Review Board at Taipei Medical University (TMU-JIRB N201806010) approved the study protocol. The study included head and neck cancer patients who presented with chronic sialadenitis, characterized by xerostomia and/or recurrent glandular pain or swelling on at least two occasions within more than three months. The eligible cancer types included nasopharyngeal cancer (NPC), oral cavity, oropharyngeal, and laryngeal cancer. A retrospective review of medical records was performed for responsiveness analysis on patients who received the therapy between November 2020 and October 2022.

A total of 147 patients who presented with persistent xerostomia and recurrent salivary glandular swelling for more than three months and received at least one round of salivary ductal irrigation were included in this study. Patients with underlying NPC constituted the main population of this study (*N* = 103), and the remaining patients were assigned to the non-NPC head and neck cancer (or shortly called as non-NPC HNC, *N* = 32) group or the non-cancer sicca syndrome group (*N* = 12). Patients with history of failure to identify or cannulate their salivary duct, or patients without identifiable records in terms of treatment response were excluded. In addition, five other patients with underlying post-radioiodine sialadenitis or obstructive sialadenitis were excluded.

### 2.2. Salivary Gland Ductal Irrigation

Patients were instructed to observe oral hygiene and fast for one hour prior to the irrigation treatment. Unstimulated whole-mouth saliva was initially collected via spitting method in order to measure salivary amylase levels just before each therapy session. The irrigation procedure involved positioning the patient in a seated position and administering 10% xylocaine over the bilateral buccal and sublingual areas. Following dilation of ductal orifices through serial conical dilators (size 1 and 2, Karl Storz, Tuttlingen, Germany), a 24# intravenous catheter was inserted into the duct, with subsequent administration of 1 mL of 0.5% dexamethasone (Standard Chem. & Pharm. Co., Ltd., Tainan, Taiwan) under light pressure for irrigation purposes. Any sign of ductal stenosis or obstruction was reflected by reciprocal resistance observed during this process. After irrigation, glandular massage was performed for ten seconds followed by removal of the catheter; the frequency of irrigations of once per month was determined based on clinical symptoms and therapeutic response as established by previous studies which employed an intraductal volume of 1 mL per irrigation session as standard protocol.

### 2.3. Definition of Responsive and Non-Responsive

While sialometry provides valuable information about saliva production, it is important to note that the objective measures may not always align perfectly with patients’ subjective experiences. Morita et al. [19] identified that about 20% and 40% of the participants from community-dwelling older adults in Japan presented with a low unstimulating or low stimulatory salivary flow rate, respectively, but without xerostomia. Similar discrepancies have also been reported previously. In addition, the assessments of sialometry may not capture other important aspects of xerostomia such as changes in the salivary constituents, oral discomfort, difficulty in swallowing, and impact on quality of life. Thus, this study aimed to interpret any difference between responsiveness and non-responsiveness to the treatments from patients’ perspectives. In this study, the criteria for responsiveness and non-responsiveness regarding the effectiveness of salivary gland ductal irrigation therapy for head and neck cancer patients were defined as follows:

Non-responsive: The patient’s medical records showed no improvement in symptoms after receiving salivary gland ductal irrigation therapy, and they only underwent the therapy once without any positive outcomes.

Responsive: The patient’s medical records demonstrated an improvement in symptoms related to chronic sialadenitis after undergoing salivary gland ductal irrigation therapy, and they received multiple treatment sessions due to a positive response to the initial therapy.

In addition to the information retrieved from the medical records, the number of treatment sessions received by each patient was also documented and used as a criterion for defining responsiveness, as non-responsive patients were typically not recommended to be further treated with ductal irrigation, and responsive patients should return on a monthly basis until the desired treatment is completed.

### 2.4. Statistical Analysis

Descriptive results were presented as absolute numbers, percentages, or mean ± standard deviation (S.D.). Intergroup comparison was carried out with the Chi-square test or one-way ANOVA test for nominal or numerical variables. Responsiveness to the treatments in each disease group and in different stages in the NPC subgroup was demonstrated using bar charts. Logistic regression analysis was conducted to explore the influence of age, gender, disease category, stage, and unstimulated saliva amylase level at the first visit on treatment responsiveness. The results were presented as odds ratios and 95% confidence intervals. Furthermore, baseline salivary amylase levels were compared to those post-first irrigation treatment to search for any predictive values by using the following methods: paired sample *t*-tests assessed improvement, and independent *t*-tests compared fold changes between responsive and nonresponsive groups in the same population. The ratio was defined as post-treatment salivary amylase level divided by pre-treatment measurement for each individual participant in this study. A *p*-value of less than or equal to 0.05 was considered statistically significant.

## 3. Results

### 3.1. Patient Characteristics and Treatment Response

**Figure 1:**
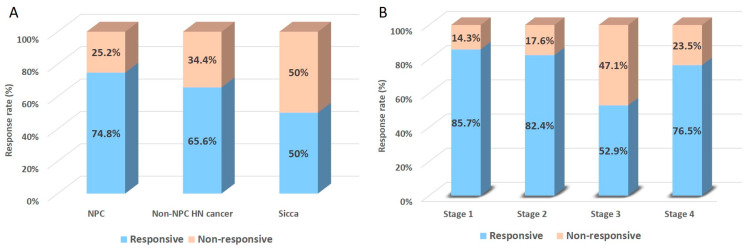
General response rates to salivary gland ductal irrigation (**A**) in each disease category and (**B**) in different disease stages in NPC patients.

Between the two-year interval from November 2020 to October 2022, a total of 147 patients were included as follows: NPC: 103 patients; non-NPC HNC: 32 patients; and sicca syndrome: 12 patients. All patients received at least one round of successful salivary ductal cannulation and irrigation. Being able to identify the stage of disease from medical records was shown to have a higher propensity for advanced-stage patients (stage III and IV) (Table 1). Then, patients in each disease entity were divided into two groups based on their treatment responses. Figure 1A reveals that the response rates for the NPC, non-NPC HNC, and sicca syndrome groups were as 74.8%, 65.6%, and 50%, respectively, without significant differences (intergroup comparison: NPC versus sicca, *p* = 0.09; NPC versus non-NPC HNC, *p* = 0.37; non-NPC HNC versus non-cancer sicca, *p* = 0.49). All stages of patients within the NPC group demonstrated favorable responsiveness after salivary ductal irrigation, except for the stage III NPC patients who showed modest improvement rates (Figure 1B) (intragroup comparison, *p* = 0.08).

**Figure 2:**
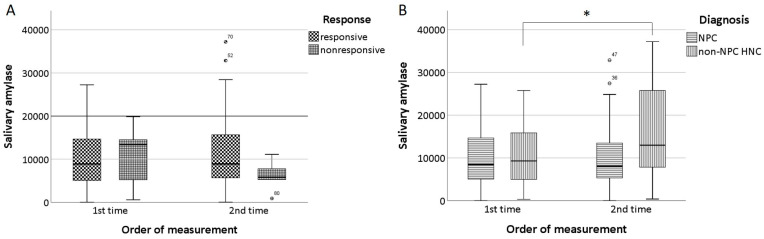
Comparison of the changes in salivary amylase level upon first and second outpatient visit (**A**) in responsive and non-responsive group; (**B**) in responsive NPC and responsive non-NPC HNC subgroups. * indicates *p* < 0.05.

Among responsive patients in each disease group, the mean number of treatments for salivary ductal irrigation were 3.6 ± 1.0, 3.8 ± 0.9, and 3.3 ± 1.0 in the NPC, non-NPC HNC, and non-cancer sicca syndrome groups, respectively (Table 1), with no significant differences being observed (*p* = 0.54). The time intervals between successive irrigation therapies among responsive patients in each disease group also yielded similar results, ranging from four to six weeks. Upon comparison of the absolute value of baseline salivary amylase levels with those measured after initial irrigation therapy, paired *t*-tests did not reveal any significant elevations or time-dependent changes in the head and neck cancer patients overall (Table 2, Figure 2A). However, post-treatment amylase levels were found to be significantly higher than pre-treatment levels in responsive non-NPC head and neck cancer patients (as outlined in Table 2, and Figure 2B), while no differences were demonstrated in the responsive NPC patients. On the other hand, an interpretation of the treatment differences based on the ratio of post-treatment to pre-treatment amylase levels still revealed no statistically significant differences between responders and non-responders across all of the tested patients as well as within the NPC subgroup (Table 2).

### 3.2. Factors Associated with Treatment Responsiveness

A logistic regression analysis was conducted to ascertain the impact of age, gender, disease category, disease stage, and initial saliva amylase level on treatment responsiveness. The results indicated that none of these factors had a statistically significant effect on ductal irrigation therapy’s effectiveness. However, it is worth noting that the NPC patients exhibited a borderline positive response to the treatment (odds ratio = 3.10; 95% confidence interval: 0.81–11.88; *p* = 0.099) (Table 3).

## 4. Discussion

The results of this study revealed a favorable overall response rate for post-irradiated head and neck cancer patients with chronic sialadenitis and xerostomia, with the majority of patients experiencing subjective improvements in their symptoms after the salivary ductal irrigation therapy. This finding suggests that the alternative irrigation therapy holds promise as an effective intervention for managing intractable xerostomia in this patient population. Although logistic regression analysis found no explicit indicators for predicting positive outcomes, the 74.8% response rate and the borderline significance in the NPC group may indicate a better performance of the irrigation therapy in this type of head and neck cancer.

The study addresses a crucial gap in treating xerostomia in post-irradiation head and neck cancer patients, a condition that severely affects their quality of life. Traditional treatments often fall short as they do not tackle structural changes within the salivary glands. This research explores the effectiveness of salivary ductal steroid irrigation coupled with ductal dilatation, focusing on both subjective symptom improvement and objective measures of gland function through salivary amylase levels. The study provides essential insights that could significantly influence clinical practices and enhance patient care outcomes by investigating a novel treatment modality not extensively documented for this specific patient population.

The management of xerostomia in post-irradiated head and neck cancer patients is a challenging task. These patients often experience significant discomfort and a reduced quality of life due to their dry mouth symptoms. Therefore, identifying effective treatment strategies is crucial for improving their well-being. While the prevalent intensity-modulated radiation therapy (IMRT) technique was proven to effectively reduce xerostomia [20,21], there were still variable proportions of post-irradiated patients that presented with symptoms or complaints relating to chronic dry mouth or hyposalivation. To tackle this issue, sialagogues stimulations and artificial saliva may be attempted, but they only offer temporary relief because of their short duration of action and necessitate repeated application for long-term satisfaction [22]. Parasympathomimetic drugs like pilocarpine or cevimeline stimulate salivary secretion, but the systemic side effects they cause may lead to intolerance and discontinuation of use [8]. The findings of our study provide valuable insights into the therapeutic efficacy of this novel irrigation treatment and its potential role in managing xerostomia beyond the disease entity of Sjogren syndrome. In addition, by understanding the factors that influence treatment responsiveness, clinicians can better select appropriate therapeutic options for individual patients, thereby optimizing treatment outcomes.

Salivary gland ductal irrigation is a therapeutic procedure involving the flushing of the salivary ducts with a variety of solutions to address conditions such as obstructive sialadenitis, chronic recurrent parotitis, and xerostomia. Saline solution, frequently utilized for its safety and non-irritating nature, mechanically removes debris [10,11,17]. Antibiotics like gentamicin [23] are directed towards bacterial pathogens, particularly in cases of sialadenitis or post-operative infections, although their inappropriate use can result in resistance and allergic responses. Corticosteroids such as dexamethasone are efficacious in reducing inflammation in chronic conditions like Sjögren’s syndrome [13,14,15,16], but come with potential systemic effects when used over an extended period. Lastly, analgesics such as lidocaine [24] offer transient pain relief during procedures, and are crucial for managing discomfort, yet carry a risk of toxicity if excessively used. Each solution is selected based on the individual patient’s specific requirements and the underlying condition, necessitating thorough evaluation by healthcare professionals.

To the best of our knowledge, this was the first study that investigated the efficacy of salivary ductal irrigation therapy in managing xerostomia mainly in post-irradiated head and neck cancer patients. Our findings are in line with several previous studies. Aframian et al. [25] analyzed one hundred patients suffering from salivary gland secretory dysfunction (including eight patients status post receiving head and neck irradiation and nineteen patients with radioiodine-related sialadenitis) and revealed improvement in unstimulated whole salivary flow (UWSF) after series of intraductal irrigation followed with sialendoscopy irrigations. The average number of times that irrigation therapy was carried out through the ordinary ductal or sialendoscopic route in their study were 6.87 ± 4.67 and 5.35 ± 3.20 times, respectively. In addition, a sustained long-term improvement in UWSF for an average of 2.33 ± 2.07 months after the sialendoscopic irrigation was achieved. Furthermore, the analysis of between-subject effects on UWSF measurements in their study showed statistically significant results for the use of a salivary gland manipulator as a negative prognostic factor, which corresponded to our previously published study [18] which identified anti-xerostomic medicine as a significant indicator for irresponsiveness to the irrigation treatment. Another study from the same institute [17] also demonstrated similar findings, and identified that different diagnosis/etiologies for dry mouth had no significant associations with subjective or objective improvements after irrigation therapies, which was similar to our results. Douglas et al. [26] reported 88.1% of symptomatic resolution in a total of 59 patients with a history of either Sjogren’s syndrome or post radioactive iodine therapy status after a corticosteroid injection and a ductal dilation in sialendoscopic surgery, and again disclosed no significant predictors of the ultimate success rates of sialendoscopy in these patient populations.

The results of our study have important clinical implications for the management of xerostomia in post-irradiated head and neck cancer patients. By demonstrating the effectiveness of salivary gland ductal irrigation therapy, clinicians can consider this treatment modality as a viable option in this disease population. Increased oral wetness may further enhance compliance with further treatments and overall quality of life. In addition, no obvious predictors or indicators were identified relating to the treatment responsiveness from either our study or the previous literature [25,26], which implies that salivary ductal irrigation therapy may have the potential to prevent wide populations being afflicted with xerostomia or repeated salivary glandular swelling, and the success rate is irrelevant to their underlying illness.

A noteworthy observation from our research was that a larger proportion of patients diagnosed with nasopharyngeal carcinoma exhibited a greater propensity to derive benefits from salivary irrigation therapy when compared to individuals suffering from other forms of head and neck cancer. Since lymph node metastasis and recurrence for NPC are relatively rare in the 1b area of the neck [27], the submandibular area was generally not covered within the target volume of radiation to prevent further xerostomia [28]. Furthermore, in cases of advanced head and neck cancer beyond nasopharyngeal carcinoma (NPC), the unilateral submandibular gland is commonly removed alongside lymphoadipose tissue from level 1b during a traditional neck dissection. By avoiding excision or damage to the submandibular gland through high-dose radiation treatment for NPC patients, salivary flow that is not stimulated can be significantly preserved, resulting in an improved quality of life.

The previous literature has shown that serum amylase may be capable of reflecting acute or chronic salivary dysfunction in head and neck cancer patients undergoing radiotherapy [29,30]. While serum amylase comprises salivary and pancreatic amylase with near-equal proportions [31], measurements of the concentration of amylase directly from saliva seems to be able to represent the salivary gland function without the interference of pancreatic lesions. Nevertheless, our findings did not establish a robust correlation between the degree of increase in the levels of salivary amylase and the efficacy of irrigation treatments. Although an increase in this value is expected to be observed in responsive patients, our study only demonstrated a correlation with the ratio of the increase in post-treatment salivary amylase levels in non-NPC HNC patients. However, this may be due to the small number of subjects who completed the amylase test in our study, leading to significant bias. Additionally, there are several factors that could account for the limited utilization of salivary amylase levels including a lack of direct sampling from ducts for measurements and potential dilution due to increased flow and fluid volume. Nonetheless, we observed a fluctuating increase in post-treatment salivary amylase levels which suggests that this marker may have clinical value, making it worthy of further exploration and clarification.

Despite our efforts, we were unable to establish a correlation between symptomatic improvements resulting from irrigation therapy and any other clinical parameters. One cohort study based on two Swedish resident populations revealed that the prevalence of xerostomia was common in older people, especially at night and in women. The reported prevalence of daytime xerostomia in women and men increased from 23.3% and 14.7% at age 50 to 39.5% and 27.5% at age 75, respectively [32]. Another study further estimated that the average yearly incidence of dry mouth after age 50 was 0.99–3.28% [33]. Their findings suggest that being female or above the age of 50 may serve as indicators for the likelihood of developing xerostomia. Regrettably, our investigation failed to reveal any noteworthy factors that could be utilized in forecasting the therapeutic results of salivary ductal irrigation. We posit that the absence of significance in our findings may be attributed to the wide age range and pronounced gender imbalances among our three disease cohorts. Nonetheless, given that no prognostic variables were uncovered in our analysis, further examination is warranted. Consequently, salivary ductal irrigation may serve as a viable alternative for all suitable head and neck cancer xerostomia patients if traditional xerostomia therapy proves inadequate.

Despite the valuable insights provided by this study, several limitations need to be acknowledged. Firstly, the retrospective nature of the study design introduces inherent biases and limitations associated with data collection and analysis. The reliance on medical records for data extraction may result in incomplete or missing information, which could potentially impact the accuracy of the findings. Additionally, the retrospective design limits our ability to establish causality between the treatment and the observed outcomes. Prospective studies with well-defined protocols are warranted to confirm the effectiveness of salivary gland ductal irrigation therapy in managing xerostomia. Secondly, the sample size used in our study was relatively small, which may limit the generalizability of the findings. A larger sample size would enhance the statistical power and provide more robust conclusions. Furthermore, the study was conducted at a single center, which may introduce institutional-specific factors that could influence the results. Multi-center collaborations would help validate the findings and enhance the generalizability of the results. Finally, the follow-up period in our study was relatively short, and the long-term outcomes of salivary gland ductal irrigation therapy were not assessed. Further studies with longer follow-up periods are needed to evaluate the durability of treatment response and the long-term effects on salivary gland function and patient-reported outcomes.

It is important to acknowledge the absence of a control group using steroid-free irrigations in this study. Saline-only irrigation may contribute to the dilation of ducts or the removal of mucus plugs, potentially leading to the restoration of gland function. Nevertheless, due to the enduring damage caused by radiotherapy to salivary glands and ducts, corticosteroids could offer additional benefits by reducing inflammation. This is corroborated by research conducted on patients with Sjogren’s syndrome or recurrent sialadenitis. Moreover, given the common use of steroid irrigation in sialendoscopy, our study deliberately excluded a cohort that received non-steroid treatments, leaving room for exploration in future investigations.

The findings of this study open avenues for future research and exploration. One important aspect for further investigation is understanding the mechanisms underlying the relationship between disease category and treatment responsiveness. For instance, while radiation-induced hyposalivation mainly affect acinar cells possibly through aberrant calcium signaling, increased reactive oxygen species production or DNA damage [34], the chiefly affected part in patients with radioiodine related sialadenitis is the luminal ductal cells that contain the sodium/iodide symporters [17]. Elucidating the molecular and physiological factors involved in the response to salivary gland ductal irrigation therapy can provide valuable insights into personalized treatment strategies. Additionally, studies focusing on long-term outcomes, including the sustainability of treatment response and impact on patients’ quality of life, are warranted. Further refinement of the technique, optimization of irrigation protocols, and identification of potential adjunctive therapies can also enhance the efficacy of salivary gland ductal irrigation in managing xerostomia.

Furthermore, the integration of patient-reported outcomes and quality-of-life assessments into future studies will provide a comprehensive understanding of the impact of salivary gland ductal irrigation therapy on patients’ well-being. This will facilitate shared decision-making between patients and healthcare providers and enable treatment plans that align with patients’ individual goals and preferences.

## 5. Conclusions

In conclusion, our study demonstrated the effectiveness of salivary gland ductal irrigation therapy in improving symptoms related to chronic sialadenitis in post-irradiated head and neck cancer patients with xerostomia. Though disease category was not identified as a significant predictor of responsiveness to irrigation therapy, future prospective studies with larger sample sizes, longer follow-up periods, and multi-center collaborations are warranted to confirm our findings and establish evidence-based treatment approaches for alleviating the burdens of xerostomia in this disease population and optimizing long-term benefits.

## Figures and Tables

**Table 1:** Demographic variables and measures of salivary ductal irrigation.

	Patient Group					
	NPC (*N* = 103)		Non-NPC HN Cancer (*N* = 32)		Sicca Syndrome (*N* = 12)	
Age (years) ^1^	47.5 ± 9.9		58.0 ± 9.7		60.1 ± 13.7	
Gender (M:F) ^2^	65:38		23:9		2:10	
Number of patients per stage						
Stage I	7		1			
Stage II	17		2			
Stage III	34		6			
Stage IV	17		10			
	**NPC**		**Non-NPC HN Cancer**		**Sicca Syndrome**	
	**Responsive**	**Non-Responsive**	**Responsive**	**Non-Responsive**	**Responsive**	**Non-Responsive**
Number of patients	77	26	21	11	6	6
Number of irrigation treatments	3.6 ± 1.0	1.2 ± 0.5	3.8 ± 0.9	1.0 ± 0.0	3.3 ± 1.0	1.0 ± 0.0
Stage I	3.3 ± 0.8	1	NA	NA		
Stage II	4.1 ± 0.9	1	NA	NA		
Stage III	3.4 ± 1.0	1.3 ± 0.5	4.3 ± 1.7	1.0 ± 0.0		
Stage IV	3.6 ± 1.0	1	3.6 ± 0.5	NA		
Interval of irrigation treatments (week)	6.2		4.9		4.4	

^1^ One-way ANOVA with post hoc Scheffe test: NPC vs. non-NPC, *p* < 0.001; NPC vs. sicca, *p* < 0.001; non-NPC vs. sicca, *p* = 0.834. ^2^ Chi-square test with Fisher exact test for inter-group gender comparison: *p* = 0.003. NA: cannot be analyzed due to scarcity of patients.

**Table 2:** Measures of salivary amylase before and after initial salivary ductal irrigation treatment.

	Amylase 1 ^a^	Amylase 2 ^a^	Paired *t* Test			$Fold=\frac{Amylase\;2}{Amylase\;1}$	Independent *t* Test		
			*t* Value	*df*	Sig (two-Tailed)		*t* Value	*df*	Sig (Two-Tailed)
**Total**									
Responsive (*N* = 35)	11,048 ± 1412	13,213 ± 2479	−0.94	34	0.353	1.42 ± 0.19	1.17	38	0.25
Nonresponsive (*N* = 5)	10,713 ± 3452	6175 ± 1670	1.82	4	0.143	0.84 ± 0.22			
	**Amylase 1 ^a^**	**Amylase 2 ^a^**	**Paired *t* Test**			$Fold=\frac{Amylase\;2}{Amylase\;1}$	**Independent *t* Test**		
			***t* Value**	***df***	**Sig (two-Tailed)**		***t* Value**	***df***	**Sig (Two-Tailed)**
**NPC**									
Responsive (*N* = 28)	10,178 ± 1347 ^b,c^	12,727 ± 2928	−0.98	27	0.338	1.28 ± 0.18	1	31	0.325
Nonresponsive (n = 5)	10,713 ± 3452 ^b^	6175 ± 1670	1.82	4	0.143	0.84 ± 0.22			
**Non-NPC HNC**									
Responsive (*N* = 7)	11,018 ± 3509 ^c^	16,838 ± 4986	−3.26	6	0.017 *	1.97 ± 0.55			

^a^ “Amylase 1” refers to baseline salivary amylase before the first time of irrigation treatment; “Amylase 2” means the value after first irrigation therapy. ^b^ Comparison of salivary amylase 1 in NPC patients between responsive and nonresponsive subgroup by independent *t* test: *p* value = 0.878 (two-tailed). ^c^ Comparison of salivary amylase 1 between responsive NPC patients and responsive non-NPC HNC subgroup by independent *t* test: *p* value = 0.793 (two-tailed). * indicates *p* < 0.05.

**Table 3:** Binary logistic regression analysis for responsiveness of salivary ductal irrigation.

Factor	OR	95% CI		*p* Value
Age	0.99	0.96	1.03	0.694
Gender	0.76	0.34	1.67	0.488
Disease category				0.245
NPC	3.10	0.81	11.88	0.099
non-NPC HNC	2.20	0.53	9.15	0.277
Disease stage	1.03	0.64	1.67	0.896
Salivary amylase level at the first visit	1.00	1.00	1.00	0.548

OS = odds ratio; 95% CI = 95% confidence interval.

## Data Availability

Data are contained within the article.
